# Impaired Cortical Tracking of Speech in Children with Developmental Language Disorder

**DOI:** 10.1523/JNEUROSCI.2048-23.2024

**Published:** 2024-04-08

**Authors:** Anni Nora, Oona Rinkinen, Hanna Renvall, Elisabet Service, Eva Arkkila, Sini Smolander, Marja Laasonen, Riitta Salmelin

**Affiliations:** ^1^Department of Neuroscience and Biomedical Engineering, Aalto University, Espoo FI-00076, Finland; ^2^Aalto NeuroImaging (ANI), Aalto University, Espoo FI-00076, Finland; ^3^BioMag Laboratory, HUS Diagnostic Center, Helsinki University Hospital, Helsinki FI-00029, Finland; ^4^Department of Linguistics and Languages, Centre for Advanced Research in Experimental and Applied Linguistics (ARiEAL), McMaster University, Hamilton, Ontario L8S 4L8, Canada; ^5^Department of Psychology and Logopedics, University of Helsinki, Helsinki FI-00014, Finland; ^6^Department of Otorhinolaryngology and Phoniatrics, Head and Neck Center, Helsinki University Hospital and University of Helsinki, Helsinki FI-00014, Finland; ^7^Research Unit of Logopedics, University of Oulu, Oulu FI-90014, Finland; ^8^Department of Logopedics, University of Eastern Finland, Joensuu FI-80101, Finland

**Keywords:** development, developmental language disorder, machine learning, magnetoencephalography, speech processing

## Abstract

In developmental language disorder (DLD), learning to comprehend and express oneself with spoken language is impaired, but the reason for this remains unknown. Using millisecond-scale magnetoencephalography recordings combined with machine learning models, we investigated whether the possible neural basis of this disruption lies in poor cortical tracking of speech. The stimuli were common spoken Finnish words (e.g., dog, car, hammer) and sounds with corresponding meanings (e.g., dog bark, car engine, hammering). In both children with DLD (10 boys and 7 girls) and typically developing (TD) control children (14 boys and 3 girls), aged 10–15 years, the cortical activation to spoken words was best modeled as time-locked to the unfolding speech input at ∼100 ms latency between sound and cortical activation. Amplitude envelope (amplitude changes) and spectrogram (detailed time-varying spectral content) of the spoken words, but not other sounds, were very successfully decoded based on time-locked brain responses in bilateral temporal areas; based on the cortical responses, the models could tell at ∼75–85% accuracy which of the two sounds had been presented to the participant. However, the cortical representation of the amplitude envelope information was poorer in children with DLD compared with TD children at longer latencies (at ∼200–300 ms lag). We interpret this effect as reflecting poorer retention of acoustic–phonetic information in short-term memory. This impaired tracking could potentially affect the processing and learning of words as well as continuous speech. The present results offer an explanation for the problems in language comprehension and acquisition in DLD.

## Significance Statement

The neural basis of impaired speech processing in developmental language disorder (DLD) was probed with magnetoencephalography, natural spoken words and sounds, and state-of-the-art machine learning models. Cortical tracking of speech was normal at initial stages but impaired at syllable-level latency, reflecting problems in maintaining cortical memory representations of the incoming speech across the word. This offers an explanation for the problems in DLD in identifying words and learning new ones.

## Introduction

Developmental language disorder (DLD) is a failure of normal language development despite adequate learning environment and lack of intellectual or physical disability (Leonard, 2014; Bishop et al., 2017). It involves deficits in varying, often multiple, aspects of language development, and theories of its causes range from “high-level” deficits to hypothesized problems in “low-level” sensory processing. Also, the specificity of problems to speech processing and the existence of a more general auditory deficit have been debated. According to one hypothesis, the root cause for DLD might lie in deficient processing of auditory information, i.e., in perceiving duration and frequency cues that are crucial for discrimination of phonemes and words. Some brain imaging studies have shown difficulties in discriminating sounds, but results are inconsistent (review Bishop, 2007). Brain imaging of DLD is scarce, and a broad view of the underlying cortical causes of the disorder is still missing. A comprehensive account of DLD should explain the problems in language acquisition ranging all the way from learning to repeat single words to understanding grammatically complex sentences.

Recently, cortical tracking of the amplitude modulations of speech has emerged as a potential mechanism for mapping between the different levels of linguistic processing (phonemes, words, sentences) and the acoustic signal and parsing the speech stream into linguistic units (Ding and Simon, 2014; Ding et al., 2016; Obleser and Kayser, 2019). Amplitude modulations are important for speech intelligibility (Shannon et al., 1995), and their cortical tracking has been shown to be especially prominent for speech, in contrast to other sounds with comparable amplitude fluctuations (Nora et al., 2020; Zuk et al., 2021). Amplitude envelope tracking reflects the tracking of multiscale spectrotemporal features that are synchronized to the overall rhythm of speech (Obleser et al., 2012; Peelle et al., 2012; Ding et al., 2014) and seems to rely on the encoding of acoustic edges (amplitude change, e.g., at syllable onsets) in evoked responses (Oganian and Chang, 2019; Oganian et al., 2021). Based on behavioral work, children with DLD have decreased sensitivity to amplitude envelope modulations (prosody, amplitude rise times; Coady and Evans, 2008; Beattie and Manis, 2013; Cumming et al., 2015; Richards and Goswami, 2015), pointing to a potential deficit in the cortical processing of these aspects of speech. During development, sensitivity to the speech amplitude modulations and their cortical tracking have been suggested to be important not only for learning to extract phonological information but also for the acquisition of morphology and syntax (temporal sampling theory; Goswami, 2019, 2022; Leong and Goswami, 2015).

Impairments in cortical tracking of speech amplitude envelope have been widely studied as a possible underlying mechanism for phonological impairments in developmental dyslexia (Abrams et al., 2009; Molinaro et al., 2016; Power et al., 2016; Di Liberto et al., 2018; Keshavarzi et al., 2022; Palana et al., 2022). Somewhat surprisingly, results relating to cortical tracking of speech in adults or children affected with DLD have not yet been published. Dyslexia and DLD are overlapping disorders, and children diagnosed with DLD early on may later be classified as dyslexics at school age, although they also have problems with a range of nonphonological language tasks (McArthur et al., 2000; Fraser et al., 2010; Ramus et al., 2013). Similar but even more prominent problems in cortical tracking of the amplitude envelope of speech could be expected in DLD, as compared with dyslexia.

Here, as a first step toward uncovering potential differences in tracking of speech in DLD, we focus on individual words and “bottom-up” tracking of acoustic and phoneme information. We build on the paradigm used in our recent study on adults, showing that the detailed cortical tracking of acoustic features supports their instantaneous transformation into linguistic representations during speech processing (Nora et al., 2020). Cortical activation was measured with magnetoencephalography (MEG) and cortical representations of spoken words investigated with machine learning models. Decoding of time-varying acoustic–phonetic features was performed using a convolution model (Faisal et al., 2015), which models the activation of neuronal populations as following the sequence of stimulus features in time. Alternative regression models with no such tracking were tested as well. In addition, we modeled cortical activation related to processing the phoneme content and semantics of the spoken words. Processing of common nouns and novel pseudowords was compared with listening to environmental sounds, to examine the specificity of the potential auditory processing problems to speech. Based on previous studies, we expected to see impaired or temporally delayed neural tracking in DLD, especially for speech.

## Materials and Methods

### Participants

Seventeen children with DLD (mean age 12 years, 0 month) and 17 children with typically developing (TD) language (mean age 11 years, 11 months) participated in the study. Most of the participants were male (10 in the DLD group and 14 in the control group). The participants were contacted through the Helsinki longitudinal SLI (HelSLI) study, aiming to highlight the etiology and prognosis of DLD in the greater Helsinki area (Laasonen et al., 2018). The children with DLD had been diagnosed at the Helsinki University Hospital prior to school entry with expressive or receptive language disorder, according to the Finnish version of the International Statistical Classification of Diseases and Related Health Problems (ICD-10). All participants were right-handed and native Finnish speakers with monolingual background, with no history of other developmental or neurological disorders or hearing impairment. The experiments were undertaken with the understanding and written consent of each participant and guardian, according to the Declaration of Helsinki and prior approval of the Helsinki and Uusimaa Hospital Ethics Committee.

### Neuropsychological testing

All children participated in neuropsychological testing in the context of the HelSLI study at schools or at Aalto University before the MEG measurements. The test battery tapped nonlinguistic and linguistic reasoning, working memory and processing speed (Wechsler, 2010), as well as phonological processing (Korkman et al., 2008) and reading skills (Häyrinen et al., 2013). Children with nonlinguistic performance below average (performance IQ < 75) were excluded from the study. The children in the DLD group showed on average poorer performance in verbal reasoning and verbal memory measures, as well as poorer reading fluency (Table 1).

**Table 1.** Results of the neuropsychological assessment

	DLD (*n* = 17)	TD (*n* = 17)	Significance (*t* test)
Sex	10 boys, 7 girls	14 boys, 3 girls	N.a.
Age at time of MEG measurement	12 years, 0 month (min 10 years, 6 months; max 15 years, 8 months)	11 years, 11 months (min 10 years, 5 months; max 15 years, 9 months)	*p* = 0.41
Verbal comprehension index (WISC-IV)	Mean (SD) 79.8 (15.9)	Mean (SD) 101.9 (14.8)	*p* < 0.001*
Working memory index (WISC-IV)	81.8 (15.7)	102.6 (11.8)	*p* < 0.001*
Perceptual reasoning index (WISC-IV)	94.2 (12.7)	99.6 (16.5)	*p* = 0.146
Processing speed index (WISC-IV)	89.5 (17.2)	98.7 (17.7)	*p* = 0.068
Comprehension of instructions (NEPSY II)	7.1 (4.3)	10.4 (3.7)	*p* = 0.011*
Phonological processing (NEPSY II)	8.9 (2.9)	9.6 (3.2)	*p* = 0.25
Reading fluency (Lukilasse 2)	−1.2 (1.1)	0.2 (1.2)	*p* = 0.002*
Reading comprehension (Lukilasse 2)	0.7 (2.0)	1.9 (1.7)	*p* = 0.077

Neuropsychological testing was conducted prior to the MEG measurements in all participants. The children in the DLD group showed on average poorer verbal reasoning, verbal working memory, comprehension of instructions, and reading fluency, as investigated with *t* test. At least 35% of the participants with DLD also would potentially qualify for diagnosis of developmental dyslexia (reading fluency two standard deviations or more below age average).

* Statistically significant at the FDR-corrected level (*p* < 0.05).

We also asked the parents to rate the children's current language comprehension and production deficits on a scale from 0 (no deficit) to 5 (very high). For the DLD group, the ratings varied between 0 and 4, with an average rating of 1.5 (mild/moderate problems) in comprehension and 1.4 (mild/moderate problems) in production. The parents of the control group children did not report any language problems.

### Stimuli and experimental design

The spoken word stimuli were 44 nouns from various semantic categories, taken from our previous study (full list of stimuli can be obtained in the supplementary materials of Nora et al., 2020). The spoken words were composed of 2–5 syllables, with five compound words included in the stimuli. To increase the acoustic variability, the words were spoken by eight different speakers: four females and four males, two children/adolescents. The speaker set was rotated across participants. The uniqueness point (i.e., estimated time of lexical selection) occurred on average at 500 ms (range, 300–890 ms, from first to fourth syllable). Because of the highly transparent writing system of Finnish, the uniqueness point of a spoken word (point of divergence from all other words with a different word stem) corresponds to its orthographic uniqueness point, calculated here based on the same 1.5 billion-token Finnish Internet-derived text corpus that was used to create the semantic features (Kanerva and Ginter, 2014). In addition, eight pseudowords were presented (e.g., karsalassu, taapuri, teni). They were minimal pairs to real Finnish words, following Finnish phonotactic rules.

The 44 environmental sounds were high-quality sounds chosen from Internet sound libraries. We sought to include as much acoustic variability as possible. The environmental sound stimuli formed six categories: animals, human sounds, tools, vehicles, musical instruments, and others; the spoken word stimuli were the noun labels of the environmental sounds.

The sounds were modified using the Adobe Audition program to mono sounds with sampling frequency of 44.1 kHz and bit rate of 16 bits. All sounds were filtered with an 8 kHz linear low-pass fast Fourier transform (FFT) filter (Blackman–Harris) and resampled at 16 kHz. Mean amplitudes of the stimuli were normalized such that the root-mean-square power of each stimulus was the same. Stimulus duration was on average 810 ms (SD 180 ms) for the spoken words and 920 ms (SD 230 ms) for the environmental sounds.

The stimuli were delivered through a panel speaker at the level of normal conversation (50–60 dB SPL, measured inside the MEG helmet). To reach a sufficiently high MEG signal-to-noise ratio (SNR) per stimulus item, we presented each stimulus 20 times in a pseudorandom manner. Event-related fields were calculated as an average of these 20 repetitions. In the stimulus sequence, two words spoken by the same speaker or a spoken word and an environmental sound referring to the same meaning (e.g., the word cat and a cat sound) were not presented in a row.

To ensure concentration, participants performed a one-back task: they were instructed to listen carefully to each sound, think about its meaning, and respond with lifting a finger when two sounds with the same meaning were presented one after another (4% of trials). The one-back task target trials consisted of the same word spoken by two different speakers or the same environmental sound with a different acoustic form, e.g., two different kinds of dog bark, never the corresponding sound and word. Response hand was alternated between participant pairs. Additional filler items (nine spoken words and eight environmental sounds) were presented only a few times, after task trials and initial trials in each block of the sequence. The MEG responses for one-back task trials and filler sounds were excluded from the analysis.

### MEG recording

Magnetic fields associated with neural current flow were recorded with a 306-channel whole-head neuromagnetometer (Elekta Oy for 10/11 of the DLD/TD participants and MEGIN Oy for 7/6 of the DLD/TD participants; the same device design, upgraded). The sensor array consists of 102 triple sensor elements, each with one magnetometer and two planar gradiometers. The MEG signals were acquired at 1,000 Hz and hardware filtered at 0.03–330 Hz. Eye movements and blink artifacts were monitored by two diagonally placed electrodes measuring electro-oculogram (EOG) signal. The position of the participant's head within the MEG helmet was defined using five head position indicator coils. The locations of these coils, attached to the participant's scalp, were determined with respect to three anatomic landmarks (nasion and two preauricular points) with a 3D digitizer and with respect to the sensor array by briefly feeding current to the coils during the measurement. Head movements were monitored continuously (Uutela et al., 1999). The MEG measurement was conducted in 2 d and lasted for ∼35 min on each day. On both days, the stimuli were divided into eight blocks, with breaks in between.

### Anatomical MRI acquisition

Anatomical MRIs were obtained with a 3T MRI scanner (MAGNETOM Skyra, Siemens Healthineers). The scan included a three-plane localizer and a T1-weighted anatomic image. To enable attribution of MEG activation patterns to cortical loci, we coregistered the MEG data in the same coordinate system with the individual MR images.

### MEG preprocessing and source modeling

Spatiotemporal signal space separation (Taulu and Simola, 2006) and movement compensation algorithms (Uutela et al., 1999) were applied offline to the raw data using the MaxFilter software (Elekta Neuromag Oy), to remove the effects of external interference and to compensate for head movements during the measurement. The MEG data were further preprocessed with MNE-Python. The raw data were filtered to 0.1–40 Hz. To obtain an estimate of the artifact signals caused by blinks or saccades, we averaged the MEG signals with respect to transient maxima in the EOG signal, we performed an independent component analysis on this average, and we removed the corresponding magnetic field components from the raw data (Uusitalo and Ilmoniemi, 1997).

The MEG data analysis focused on the 204 planar gradiometer channels. Trials were averaged from 300 ms before to 2,000 ms after the stimulus onset. The averaged MEG responses were baseline corrected to the 300 ms interval immediately preceding the stimulus onset. The delay in the presentation of auditory stimuli was measured using an artificial ear and corrected for. On average, 19.98 ± 0.15 (mean ± SD) artifact-free epochs (trials) per stimulus were gathered in the DLD group and 19.96 ± 0.33 in the TD group (maximum was 20). To verify that the quality of the MEG data did not differ dramatically between the two participant groups, we estimated the SNR on the responses of each participant by calculating the baseline variance over trials and dividing the *z* scored and baseline corrected mean signal intensity of the sensor signals during stimulus presentation by the estimated noise (baseline variance). These obtained SNR values were then compared between participant groups. There was no significant difference (mean SNR in DLD group 18.2 and in TD group 17.4; *t*_(32)_ = 0.547; *p* = 0.29).

Machine learning analysis was conducted on sensor-level data. An estimate of the underlying cortical sources was additionally obtained for an overview of the data and for visualization purposes, using minimum norm estimates (MNEs; Hämäläinen and Ilmoniemi, 1994) with MNE-Python. For MNE analysis, the cortical surface of each participant was reconstructed from their individual MR images with the Freesurfer software (Dale et al., 1999; Fischl et al., 1999). Each hemisphere was covered with ∼5,000 potential source locations. Currents oriented normally to the cortical surface were favored by weighting the transverse currents by a factor of 0.2, and depth weighting was used to reduce the bias toward superficial sources (Lin et al., 2006). Noise-normalized MNEs (dynamical statistical parametric maps, dSPMs) were calculated over the whole cortical area to estimate the SNR in each potential source location (Dale et al., 2000). Noise covariance matrix was estimated from the 300 ms prestimulus baseline periods across all trials. For group-level visualization, the cortical surface of each participant was morphed onto Freesurfer's average cortical surface template (fsaverage).

### Acoustic, semantic, and phonemic features of the stimuli

#### Acoustic features

**Figure 1.**
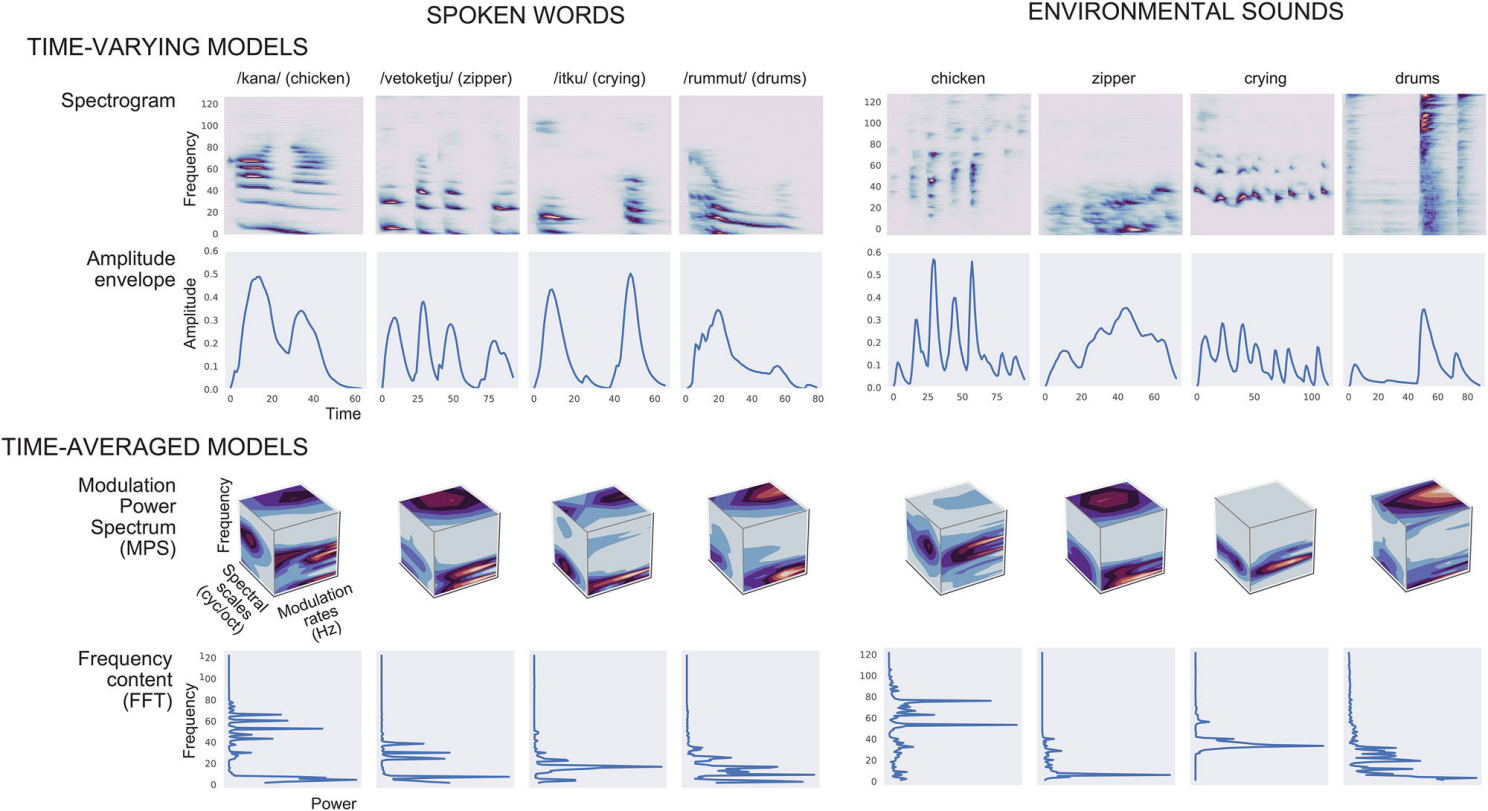
Time-varying versus nontime-varying features for modeling spoken words and environmental sounds. Visualization of the different acoustic models for example stimuli (four spoken words and the corresponding four environmental sounds).

We modeled the stimulus sounds with four sets of acoustic features. The time-varying acoustic representations that were used as features in the models were amplitude envelope and spectrogram. The amplitude envelope captures amplitude modulations within the sound but lacks fine spectral structure. In speech signals, the boundaries between syllables are encoded well by the temporal envelope. The envelope also carries information about the identity of phonemes, particularly consonants, at syllable onsets, as can be seen for the example sounds in Figure 1. The spectrogram, in turn, is a representation of the spectrotemporal fine structure of the sound (amplitude changes in the different frequency channels separately). In speech, it captures, for example, the acoustic cues responsible for formant structure of speech.

The spectrogram was created using an auditory filter bank with 128 overlapping frequency bands, mimicking the representation of sound in the human cochlea (Chi et al., 2005), with central frequencies of the bands ranging from 180 to 7,246 Hz. The sounds were divided into frames of 10 ms and integrated over 16 ms time windows. The amplitude envelope was created by averaging the sound spectrogram across the frequencies, resulting in one feature vector of the temporal changes occurring in the spectrogram. We chose to model the full range of amplitude envelope frequencies to capture the full representations of the speech signal and to allow for comparison to the other acoustic models.

For comparison, we included two nontime-varying representations of the acoustic features of the sounds. The frequency spectrum (FFT) is a nontime-varying representation of the stimulus power per frequency (the same filter bank of 128 frequency bands as for the spectrogram). In addition to displaying an organization by frequency, the primary and secondary auditory cortices respond to different rates of temporal modulations at different spectral scales (Pasley et al., 2012; Santoro et al., 2014, 2017). These modulations are captured by our second set of features, the modulation power spectrum (MPS; Chi et al., 2005; Pasley et al., 2012; Santoro et al., 2014, 2017). MPSs were calculated using the NSL toolbox (Chi et al., 2005) with modulation-selective filters spanning four spectral scales (0.5, 1, 2, and 4 cycles/octave) and four temporal rates (1, 3, 9, and 27 Hz); these have been shown to capture the essential features of a broad range of natural sounds (Santoro et al., 2014). We chose a three-dimensional MPS where the upward-going and downward-going modulations were not separated.

#### Phoneme sequence

Phonetic features represent the phoneme content within each spoken word over time. The phoneme sequences of the words were obtained using their phonemic annotation, manually time-aligned to the stimulus wavefile using the Praat software (Mesgarani et al., 2014; Di Liberto et al., 2015). Only phonemes with 10 or more instances in the stimulus set were included, resulting in a set of 15 phonemes, each occurring 10–40 times. These covered 95% of all the phonemes occurring in the familiar words and 100% of phonemes in the pseudowords. Each phoneme was set as 1 in those 10 ms time windows within the word where the phoneme was present and as −1 otherwise. Only one phoneme was marked “active” in each time window, capturing well the timing of phoneme onsets but not taking coarticulation into account.

#### Semantic features

The semantic features were obtained by concatenating two sets of norms, one acquired through a questionnaire and the other using word co-occurrences in a large-scale text corpus (Nora et al., 2020). Question norms for the stimulus words were collected with a web-based survey, where 59 university students answered 99 questions about the semantic properties of each item on a scale from 0 to 5. For extracting the corpus statistics, the frequencies of co-occurrences of words in the immediate neighborhood (five words before and five words after) of each lemmatized stimulus word were calculated from a 1.5 billion-token Finnish Internet-derived text corpus (Kanerva and Ginter, 2014).

### Machine learning models

**Figure 2.**
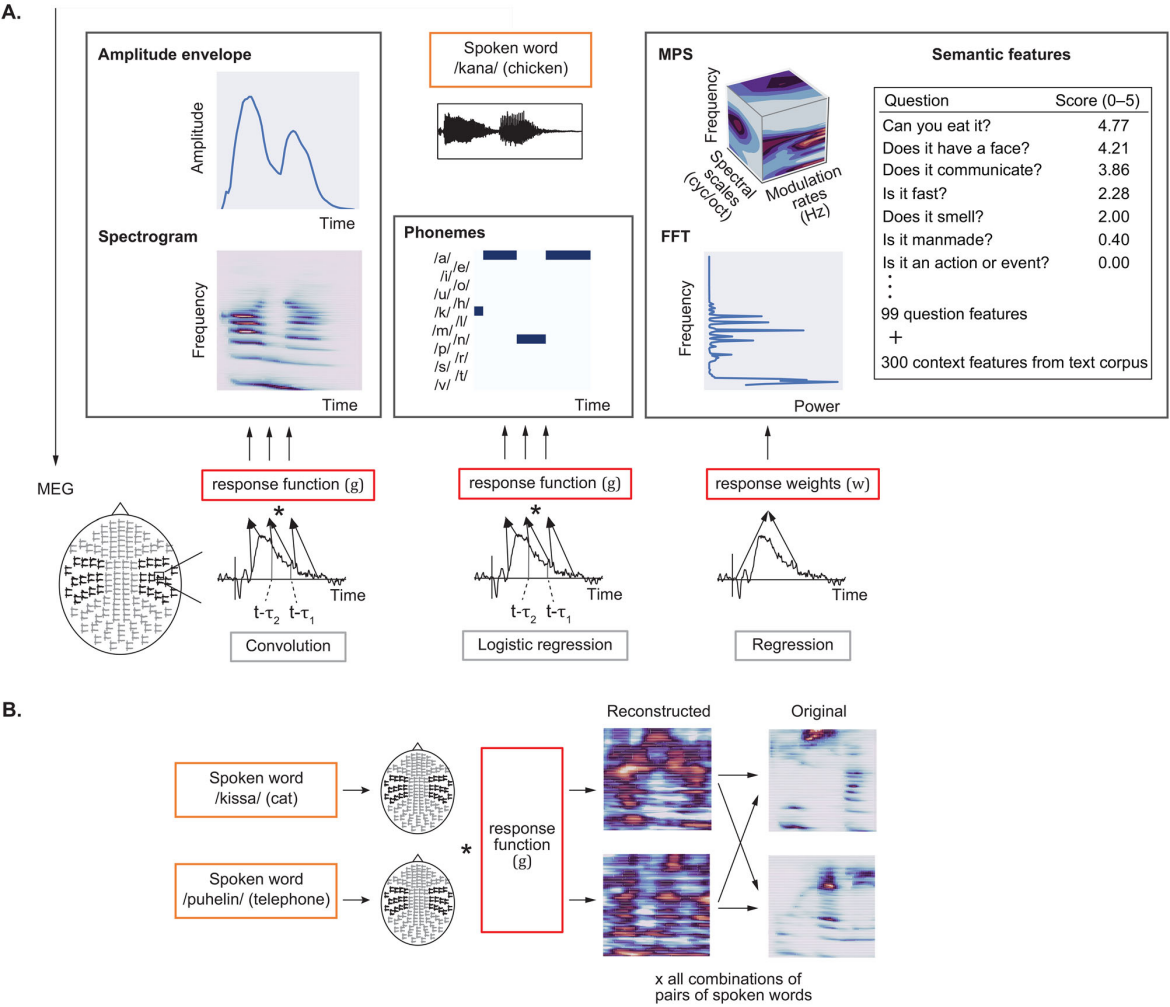
Models for decoding spoken words and environmental sounds. ***A***, Illustration of the time-locked (convolution and logistic regression) and time-averaged (linear regression) machine learning models and the different sets of acoustic, phonemic, and semantic features. The different sets of features and the different machine learning models are illustrated here for exemplary spoken words; the same models were used for decoding environmental sounds. ***B***, Visualization of the performance evaluation for the machine learning models (here for spectrogram reconstruction with the convolution model, for two spoken words).

The models that were used in predicting the different features of the stimulus sounds are illustrated in Figure 2. Each computational model aimed to learn a function *f*: *X* → *Y* that maps a set of predictive features *X* to some predicted value *Y*. Here, the predictive features *X* are derived from the observed activity in a set of MEG sensors, and *Y* is a variable indicating the value of the representation (feature) of a stimulus sound. Here, we used the success or failure of the learned function *f*: *X* → *Y* in predicting different sets of sound features *Y* in the test data to explore what kind of information is encoded in the MEG signal *X*. This decoding approach shares the basic rationale of the temporal response function (TRF) analysis (Crosse et al., 2016) in relating brain activation to different stimulus features, but it aims to reconstruct the stimulus features (e.g., the envelope) based on the MEG responses and the learned response function or response weights (backward modeling), while the TRF analysis is typically performed in the opposite direction (forward modeling).

Each decoding model was trained and tested separately for each individual participant and tested separately for the spoken words and for the environmental sounds. For acoustic and phoneme decoding, we used MEG data from sensors above the bilateral auditory cortices (28 planar gradiometer pairs). To investigate possible differences in speech processing between the two hemispheres, we additionally trained and tested separately the time-sensitive models for data from left and right auditory cortices. For decoding the semantic features, data from all 204 sensors covering the entire cortex were used.

Before performing the analyses, MEG responses were downsampled to 100 Hz (10 ms resolution). Also, all features and MEG responses were standardized across the stimuli by setting the mean value to 0 and standard deviation to 1: for the acoustic features, the FFT and spectrogram were normalized within each frequency band and the MPS within each rate, scale, and frequency band. Each semantic feature and the amplitude envelope were similarly standardized across all stimuli. The MEG signal power per sensor was normalized within each 10 ms time window. When applied to both stimulus features and corresponding MEG responses, this procedure ensures that the absolute power per frequency band is not crucial in model estimation. Instead, the unknown quantities (weights in regression and spatiotemporal response functions in convolution) are estimated such that the variation in the MEG signal power consistently correlates with variation in each stimulus feature, across stimuli.

For predicting the nontime-varying sound features (FFT, MPS, and semantic features), a simple linear regression model was used. In this model, each feature of the stimulus is reconstructed based on all time points of the MEG responses (Sudre et al., 2012), here at 0–1,000 ms after stimulus onset. For time-sensitive decoding of the acoustic features, a kernel convolution model (Faisal et al., 2015) was used. The model learns a linear mapping between the time-varying neural responses (evoked responses to sounds) and time-varying representation of the stimulus sounds (spectrogram or envelope) via convolution. This model, unlike the simple regression model, assumes that the activation of neuronal populations follows closely in time the time sequence of stimulus features. Additionally, for decoding the categorical time-varying phoneme features of the spoken words, a binary logistic regression model was used. It predicts the probability of each phoneme being active, at each time window. These models are described in detail below.

#### Convolution model for decoding time-varying acoustic features

A dual representation of a convolution model, also called a kernel convolution model (Faisal et al., 2015), was used for decoding the time-varying features. In the convolution model, a linear mapping between the neural responses, evoked by the stimulus sounds, and a time-varying representation of the original stimulus (spectrogram or envelope) is learned. We used a dual (kernel) representation of a sparse convolution model, which is practical for decoding multidimensional MEG data due to the reduced computation time (Faisal et al., 2015).

The linear mappings are learned via a convolution of the evoked neural responses r(t,x) with unknown spatiotemporal response functions g(t,f,x). More specifically, values at each frequency band of a sound are predicted at each time point *t* (moving from 0 to end of the sound) based on MEG responses (r) in the time range from $\left(t-\tau_{2}\right)$ to $\left(t-\tau_{1}\right)$, where $\tau_{1}$ and $\tau_{2}$ are the temporal lags used for the model. Thus, each time point t in the feature set is decoded based on a lagged time window in the response, and the start and end of the lag window are determined by $\tau_{1}$ and $\tau_{2}$ (Fig. 2*A*). The lag values from the stimulus to neural response are always selected to be positive, as the neural activation always follows, and never precedes, in time each time point of the stimulus that it is encoding; the model does not take into account the anticipation of the next events in the sound. This results in the prediction of the time series of amplitude changes of the sound spectrogram or the amplitude envelope. The reconstructed time series of a sound spectrogram can be modeled as follows:

(1) $${\hat{s}}_{f}(t)=\underset{x}{\sum }\;\overset{\tau =\tau_{2}}{\underset{\tau =\tau_{1}}{\sum }}\;g_{f}(\tau ,x)r(t-\tau ,x).\;(1)$$

In this model, each frequency channel of the stimulus representation is treated independently, which means that for the reconstruction of each frequency channel in ${\hat{s}}_{f}$, an independent response function $g_{f}$ is trained. Thus, for the reconstruction of one frequency channel, the mapping can be written in its matrix notation as $S_{f}=RG_{f}$ where we define $S_{f}\in R^{(NT)\times 1}$ and $G_{f}\in \;R^{(\tau x)\times 1}$ and the response matrix $R\;\in R^{\left(NT)\times (\tau x\right)}.$ Each row $r_{n}(t)$ in *R* represents the MEG response to a spoken word or environment sound *n* across all sensors *x* and all time points sampled from $\left(t-\tau_{2}\right)$ to $\left(t-\tau_{1}\right)$. Next, the unknown function $G_{f}$ is estimated by minimizing the mean-squared error between the actual $s_{f}$ and predicted the ${\hat{s}}_{f}$ representation of the stimulus sounds as follows:

(2) $$\arg\;\mathrm{mi}n_{G_{f}}\underset{n,t}{\sum }\;\{s_{f}(n,t)-{\hat{s}}_{f}(n,t){\}}^{2}+\;\lambda_{f}\underset{x,\tau }{\sum }\;g_{f}(\tau ,x{)}^{2}.\;(2)$$

Minimizing this loss function leads to the maximum-a-posteriori (MAP) estimate for $G_{f}$ as follows:

(3) $${\hat{G}}_{f}=\;(R^{T}R+\;\lambda_{f}\;I{)}^{-1}R^{T}S_{f}.\;(3)$$

This classical MAP estimate is not ideal for MEG studies where the number of conditions are typically small compared with the dimensionality of the neural responses. To solve this problem, we used the dual (kernel) representation (Faisal et al., 2015) of a convolution model. Here, the MAP estimate is obtained by replacing the inner product $R^{T}R$ with the corresponding Gram matrix $RR^{T},$ which leads to the following:

(4) $${\hat{G}}_{f}=\;R^{T}(RR^{T}+\;\lambda_{f}I{)}^{-1}S_{f},\;(4)$$

where $\lambda_{f}$ is the regularization parameter and I is an identity matrix. To estimate the regularization parameter $\lambda_{f}$, we used a grid of predefined values to find the optimal value that minimizes the leave-one-out error within the training data (Faisal et al., 2015). Given the lag parameters $\tau_{1}$ and $\tau_{2}$, the MAP estimates ${\hat{G}}_{f}$ were used to predict the time-varying features for the unseen test sounds as follows:

(5) $${\hat{s}}_{\;f\{\mathrm{TEST}\}}(t)=\underset{x}{\sum }\;\overset{\tau =\tau_{2}}{\underset{\tau =\tau_{1}}{\sum }}\;g_{f}(\tau ,x)r_{\left\{\mathrm{TEST}\right\}}(t-\tau ,x).\;(5)$$

To obtain an overview of the model's performance, we used a lag window of 0–420 ms (delay from time point in the stimulus spectrogram to a range of time points in the MEG signal, i.e., from $-\tau_{2}\;$ to $-\tau_{1}$, where $\tau_{1}=-420$ and $\tau_{2}=0$. Next, we advanced the lag window in nonoverlapping 20 ms steps (20–40 ms, 40–60 ms, …, 400–420 ms) to investigate for how long the sound features at each time point of the evolving sound are represented in the MEG responses.

#### Linear regression model for decoding time-integrated acoustic and semantic features

The time-integrated representations of the stimulus sounds (FFT, MPS, and semantic features) were decoded by using a simple linear regression model, where the unknown weight matrix $w_{f}(t,x)$ maps the neural activity *r*(*t*,*x*) at brain location *x* and time *t* to each nontime-varying feature of a stimulus sound $S_{f}.$ These nontime-varying stimulus features were decoded using MEG data from the time interval 0–1,000 ms, for the acoustic models with data from sensors over bilateral temporal cortices and for the semantic models with data from all sensor locations. The weight matrices were learned in a similar fashion as for the convolution model, by using dual representation of the regression model. Also in the linear regression model, ridge regularization was used. Using the same notation as in Equation 1, the reconstruction value ${\hat{s}}_{f}$ for one semantic or nontime-varying acoustic feature *f* can be written as follows:

(6) $${\hat{s}}_{f}=\underset{x}{\sum }\;\underset{t}{\sum }\;w_{f}(t,x)r(t,x).\;(6)$$

#### Binary logistic regression model for phoneme decoding

For the categorical phoneme representations, the linear regression model is not optimal, and a logistic regression model was used instead. For each column of phonemes $Y_{f}=[Y_{1},\ldots ,Y_{k}]\in \;R^{t\times k}$ in the phoneme feature matrix, a binary classification model was trained separately. Let p be the probability of $Y_{f}\;=1$ or p=P(Yf=1|X), which is the probability of phoneme $Y_{f}$ being active within a certain time window given the data X. In the following, the prediction framework of phoneme vector $Y_{f}$ sampled at times t=1,..,T is shown. For predicting the inactivity or activity (−1 or 1) of phoneme $Y_{f}$ at each time point t, MEG responses in the time range from τ=(τ−τ2) to (τ−τ1) were used for prediction, where τ1 and τ2 are the temporal lags. The probability p can be modeled as follows:

(7) $$P=\frac{1}{1+e^{-w^{T}X+b}},\;(7)$$

where w and b are the vectors containing the model coefficients and $X\;\in \;R^{\left(NT\times \tau x\right)}$ contain the lagged representations of the MEG responses to N sounds in the training set. To estimate the model coefficients w and b, we minimized the loss function between the predicted and observed probabilities using the stochastic gradient descent method and ridge regularization. After training the model, the estimated model coefficients $\hat{\beta }$ were used for predicting the probabilities p for onset of phoneme $Y_{f}$ in each time window by applying the sigmoid function as shown in Equation 7. If the predicted probability was larger than 0.5, the phoneme was considered active in this time window. Otherwise, the phoneme was considered inactive.

### Performance evaluation and statistical significance

The performance of all the machine learning models was evaluated using a leave-two-out cross-validation scheme (Mitchell et al., 2008), where each model learned a mapping between a stimulus feature set and the MEG response, based on all but two sounds (Fig. 2*B*). Then the model was used to predict the features based on MEG data of the two left-out sounds, and the similarity (Pearson’s correlation) between the original and reconstructed features is calculated. The decoding was considered correct if the combined similarity (sum of correlations of the reconstructed sounds to the correct originals) between the true features of sounds (*s*_1_ and *s*_2_) and the decoded features of the sound (*p*_1_ and *p*_2_) was greater than the reverse labeling (summed correlations of the reconstructed features to the features of the incorrect sounds of the test pair) as follows:

(8) $$\mathrm{sim}(s_{1},p_{1})+\mathrm{sim}(s_{2},p_{2})>\mathrm{sim}(s_{1},p_{2})+\mathrm{sim}(s_{2},p_{1}),\;(8)$$

where sim(s,p) is the Pearson’s correlation between original s and decoded features p. We additionally investigated leave-one-out reconstruction (correlation of the original and reconstructed features) for the amplitude envelope and spectrogram models; this cross-validation method yielded similar results as the leave-two-out scheme.

The performance evaluation process was repeated for all possible leave-two-out combinations: the 44 items (spoken words or environmental sounds) were divided into 42 training and 2 test sounds in all possible pairwise combinations, leading to a total of 946 pairwise tests. For the time-varying representations of the sounds, the feature vectors for the two held-out test sounds were always equalized to the length of the shorter one, to control for possible confounding effects of the varying lengths of feature vectors on the performance of the convolution model.

The final decoding accuracy of the model is the percentage of predictions that were classified as correct.

To evaluate whether the decoding accuracy for the models was statistically different from chance performance, we compared the results with those obtained from permuted data separately for each participant. In each permutation run, the item labels for the averaged evoked responses were randomly permuted across the different sounds (within spoken words or environmental sounds). This procedure was repeated 200 times for each convolution model and 1,000 times for each regression model. For each permutation, the models were evaluated using all possible pairwise combination tests in a leave-two-out cross-validation scheme. The *p* values were computed for each participant individually by calculating the number of times the permutation result was better than the observed decoding accuracy. The significance levels (*p* = 0.05) were similar for different participants (∼62% for the spoken words/environmental sounds for all models and ∼75% for pseudowords for all models); decoding performance above those levels may be considered significantly above chance-level performance, which was ∼50%. We report, for each model, the number of participants of total (*n*/17) that showed statistically significant decoding.

Comparisons between the two participant groups were performed only for the models in which the decoding accuracy reached significance in more than half (at least 9/17) of the participants in either group. Independent samples *t* tests were used for comparing the decoding performance between the two participant groups. The results were corrected for multiple comparisons over the different models tested, using the false discovery rate (FDR) at a level of 0.05.

To investigate group differences in decoding performance at different lags after sound time points (i.e., for how long information in the spoken word at each time-point is represented cortically), we used the cluster permutation testing (Maris and Oostenveld, 2007). This method is able to find clusters of time points in a time series that differ between the two participant groups, without having to choose the points manually and perform single independent tests for each time point. Thus, this method resolves the need for correcting over multiple comparisons, as it takes advantage of the fact that the decoding results in the neighboring lags are nonindependent. In this test, we investigated all lags from 20–40 to 400–420 ms. First, when testing for a difference between the two groups for each lag, we used independent samples *t* tests. We then defined clusters of lags where the difference between participants was greater than a selected threshold (e.g., alpha 0.05) with the selected test statistic and calculated the cluster mass, which is the sum of observed *t* statistics for each lag within the cluster. If there were multiple clusters, the largest one was selected, and cluster mass was calculated for that cluster only, to find the time windows with most robust group differences. We then tested whether this cluster was larger than clusters that occur by chance. This was determined by permutation testing: we created two participant groups by randomly selecting data from participants in either group and repeated the above steps, that is, calculating the cluster mass in this case. This permutation procedure was repeated 1,000 times. The *p* value for each model was obtained by calculating how many permutations produced higher cluster mass than the observed one, divided by the number of permutations [*p* value = *N* (permutation cluster mass > observed cluster mass) / *N* (permutations)]. Paired comparisons were conducted in the largest clusters identified in cluster permutation testing, as well as in a window around the lags showing the best decoding for each participant group, and effect sizes were computed for these comparisons.

### Data and code availability

Raw data were generated at Aalto University, Department of Neuroscience and Biomedical Engineering. The data are not publicly available due to ethical restrictions imposed by the research ethics committee, as brain data cannot be fully anonymized. Relevant derived and pseudonymized data supporting the findings of this study are available from the corresponding author upon reasonable request and with permission of the research ethics committee for researchers aiming to reproduce the results. Custom code used in the machine learning analysis is available from the corresponding author upon reasonable request for replication purposes.

## Results

### Behavioral one-back task performance

The DLD group successfully detected repetition of meaning for 83 ± 18% (mean ± SD) of the spoken words and 59 ± 23% of the environmental sounds; in the TD group, the hit rate was 92 ± 9% for spoken words and 66 ± 19% for environmental sounds. Overall, the participants performed better for spoken words than environmental sounds (Wilcoxon signed rank test, *Z* = 7.0; *p* < 0.001). There was no statistically significant difference between the participant groups for spoken words (Mann–Whitney *U* = 94.0; *p* = 0.080) and environmental sounds (Mann–Whitney *U* = 121.5; *p* = 0.43). To further investigate whether the one-back task performance can be considered equivalent in the two participant groups, we conducted an equivalence test with lower and upper boundaries for equivalence set to a medium-sized effect (*d* = −0.5 and 0.5). The effect size of the observed group difference for speech stimuli was *d *= 0.625, and the 90% confidence interval for the effect size was 0.048–1.204. For environmental sounds the effect size of the observed difference was *d* = 0.274, and the 90% confidence interval for effect size was −0.235 to 0.901. Thus, the performance should not be considered equivalent between the participant groups. Therefore, we sought to control for the possible contribution of attentional differences by equalizing one-back task performance (see section at the end of Results).

### Brain responses

**Figure 3.**
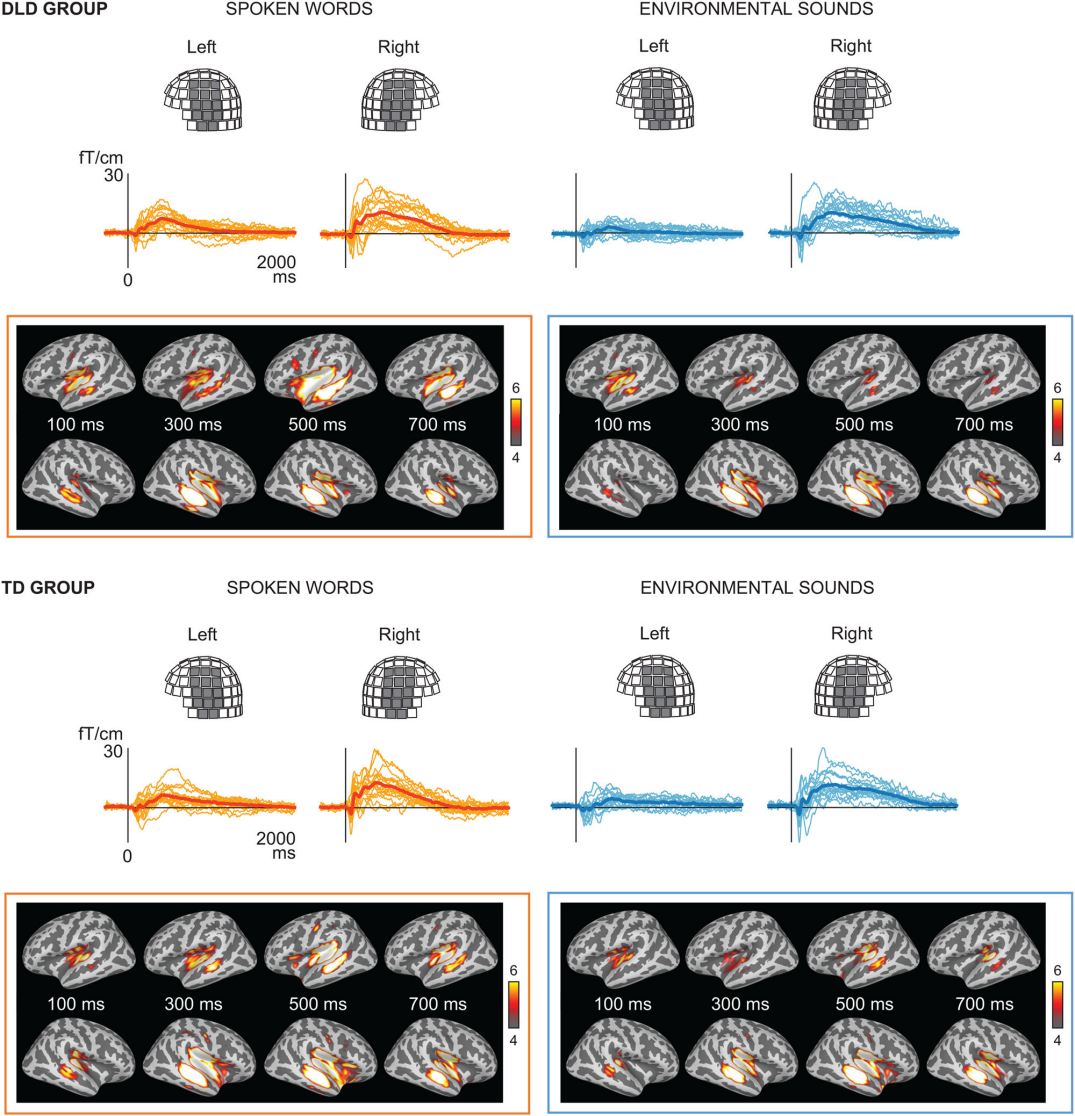
Sensor and source-level brain responses to spoken words and environmental sounds. ***A***, Grand average evoked responses averaged over all spoken words (orange) and environmental sounds (blue) and over sensors covering the left and right temporal cortices in individual participants (narrow lines) and over participants (thick lines) of the DLD and TD groups. The same selection of sensors was used for decoding of the acoustic and phoneme features in the machine learning analysis. ***B***, Cortical source maps (dSPMs) of spoken words and environmental sounds.

Responses to spoken words and environmental sounds were plotted on both sensor and source levels to get an overall view of the brain activation patterns and their timings (Fig. 3). Visually inspected, the responses in the DLD and TD groups were similar. Spoken words seemed to elicit more activation in the left temporal regions than environmental sounds in later time windows. However, right temporal responses were also prominently visible for both spoken words and environmental sounds.

### Decoding the semantic features of sounds

The environmental sounds were decoded successfully with the semantic model, with average decoding accuracy at 70% for the DLD group and 72% for the TD group. The decoding of semantic features of spoken words was near the chance level, with average decoding accuracy of 54% for the DLD group and 53% for the TD group. No statistically significant group differences were found.

### Decoding acoustic features and phoneme labels

**Figure 4.**
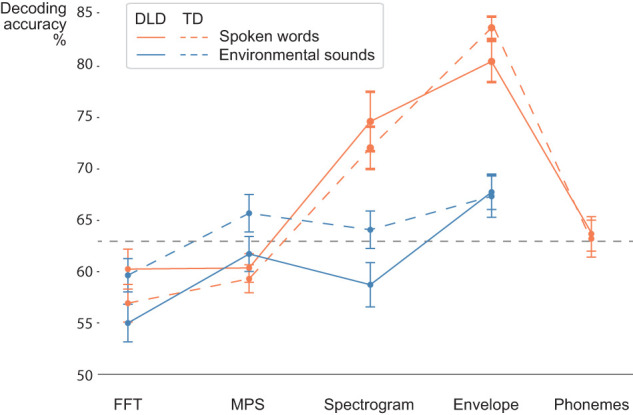
Decoding results for the different acoustic models for spoken words and environmental sounds. MEG data from sensors over bilateral temporal cortices were used in decoding. For getting an overview of decoding the amplitude envelope and the spectrogram, we used a lag from 20 to 420 ms between each time point in the stimulus features and the MEG data. The phoneme sequence of the spoken words was decoded with the same lag window using a logistic regression model. These models were compared with models using a wide time window of the MEG data (0–1,000 ms) for decoding the overall spectral content (FFT) or spectral and modulation content (MPS) for spoken words (orange) and, separately, for environmental sounds (blue). The gray solid line denotes the chance level (50%) and the gray dashed line the approximate significance level at alpha 0.05, based on permutation tests; the significance level varied somewhat between different models. The average decoding accuracy reported here means the percentage of cases, averaged across all participants, where the model finds the correct sound among two sounds, based on reconstructed features.

Figure 4 and Table 2 summarize the overall decoding differences between the different acoustic and phoneme models. For the spoken words, the time-locked acoustic models (spectrogram and amplitude envelope decoding with the convolution model) performed remarkably well. Here, the convolution model uses MEG data at 20–420 ms lag to predict each time point in the sound features. The significance limit according to permutation tests was at ∼62% across participants and models; Table 2 shows, next to the average accuracy (%) of each model, the number of participants for whom performance was significantly above the chance level. With the amplitude envelope model, the average decoding accuracy of the spoken words was at 80% (ranging from 62 to 92%) for the DLD group and 84% (ranging from 77 to 91%) for the TD group. Environmental sounds were also decoded above the chance level in most participants with the amplitude envelope model but with considerably lower accuracy (average decoding accuracy of the environmental sounds was at 68%, ranging from 57 to 77%, for the DLD group and 67%, ranging from 49 to 81%, for the TD group); decoding accuracy was higher for the speech stimuli compared with that for environmental sounds in all participants except in one participant of the DLD group. Spectrogram decoding also performed very well for the spoken words (accuracy 72–75%) and at a lower accuracy for the environmental sounds (59–64%). The average decoding accuracy for the phoneme sequence was 63% in both groups, significantly above the chance level in half or more of the participants in both groups.

**Table 2.** Average decoding results

	DLD (*n* = 17)	TD (*n* = 17)
Speech		
Speech amplitude envelope*	80% (17)	84% (17)
Speech spectrogram*	75% (14)	72% (13)
Speech phoneme sequence*	63% (11)	63% (9)
Speech FFT	60% (6)	57% (4)
Speech MPS	60% (7)	59% (4)
Speech semantic	52% (2)	53% (2)
Nonspeech		
Nonspeech amplitude envelope*	68% (14)	67% (12)
Nonspeech spectrogram	59% (5)	64% (8)
Nonspeech FFT	55% (3)	60% (5)
Nonspeech MPS*	62% (10)	66% (10)
Nonspeech semantic*	70% (14)	72% (15)
Pseudowords		
Pseudoword amplitude envelope*	75% (14)	80% (15)
Pseudoword spectrogram*	74% (9)	74% (12)
Pseudoword phoneme sequence	68% (4)	69% (5)

In the regression models a large time window of the MEG data (0–1,000 ms) and in the convolution models a wide lag window between the sound features and the MEG data (20–420 ms) were used for decoding. The numbers in parentheses show the number of participants that showed statistically significant results (*p* < 0.05) based on individually determined significance levels, calculated with permutation tests. Between-group comparisons were run for the models (marked with asterisk*) in which the decoding performance reached significance in more than half (nine or more) of the 17 participants in either participant group.

The performance was notably lower for the nontime-locked acoustic models. Only a few participants showed significant decoding of the time-averaged spectral content (FFT) of the spoken words (accuracy 57–60%) and the environmental sounds (55–60%). Also, for the time-averaged spectral and modulation content (MPS), few participants showed significant decoding for spoken words (59–60%); for the environmental sounds, decoding was somewhat better (62–66%). Between-group comparisons (performed for the models with significant decoding in more than half of the participants, marked with asterisk in Table 2) revealed no significant differences between the groups in decoding performance of any of the models after correcting for multiple comparisons.

### Cortical tracking of the acoustic envelope and spectrogram at different latencies

**Figure 5.**
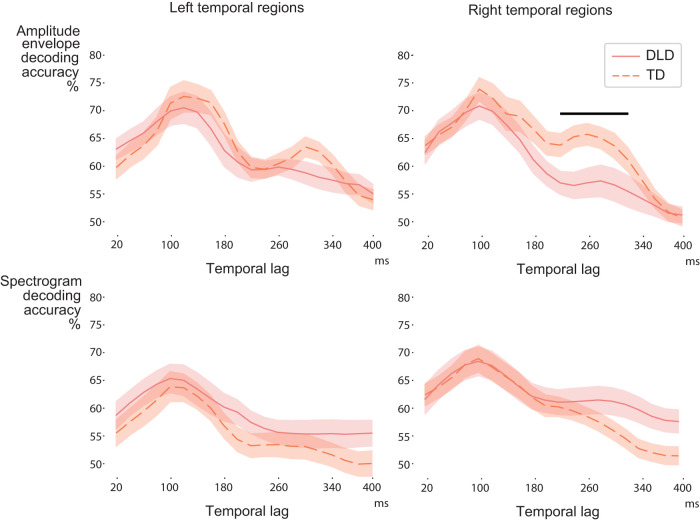
Decoding results for speech envelope and spectrogram in the left and right hemispheres in the DLD and TD groups. Solid (DLD) and dashed (TD) red lines show the average (with standard error of mean) decoding performance for each participant group. The black bar denotes the largest observed cluster showing differences (TD > DLD) based on cluster permutation testing.

To examine possible differences in the timing or duration of cortical representations between DLD and TD children, we next investigated envelope and spectrogram tracking of speech using a moving 20-ms-wide lag window and covering the lag range from 20–40 to 400–420 ms after each time point in the sound. In both participant groups, the best decoding occurred at a lag of ∼100 ms for spoken words, indicating that the acoustic information within speech is represented cortically mostly at around this latency (Fig. 5). At this lag, the decoding results were similar in the two participant groups [average decoding accuracy 76% in the DLD group and 77% in the TD group; paired comparisons of average decoding accuracies at 80–120 ms lag window (TD vs DLD): *t*_(32) _= −0.21; FDR-corrected *p* = 0.42; effect size *d* = 0.073].

However, group differences emerged in the cortical representation of amplitude envelope information at longer latencies (based on cluster permutation results in the bilateral temporal regions, with largest cluster approximately at 160–340 ms lag; *p* = 0.039), where the TD children showed higher decoding accuracy than children with DLD [average decoding accuracy 64% in the DLD group and 68% in the TD group; paired comparisons of average decoding accuracies at 160–340 ms lag window (TD vs DLD): *t*_(32)_ = 2.43; FDR-corrected *p* = 0.017; effect size *d* = 0.83]. When analyzing the data from each hemisphere separately, the group difference reached significance in the right hemisphere [largest cluster at ∼220–320 ms lag; *p* = 0.037; average decoding accuracy 57% in the DLD group and 64% in the TD group; paired comparisons of average decoding accuracies at 220–320 ms lag window (TD vs DLD), *t*_(32) _= −2.54; FDR-corrected *p* = 0.017; effect size *d* = 0.87; Fig. 5]. The spectrogram decoding also showed tentative group differences in the right temporal region in the cluster-based permutation testing (largest cluster at 300–420 ms lags; DLD > TD; *p* = 0.055).

**Figure 6.**
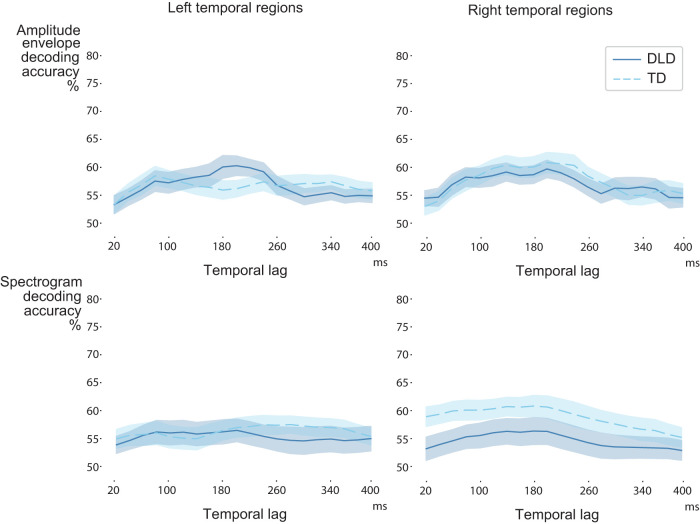
Decoding results for the nonspeech envelope and spectrogram in the left and right hemispheres in the DLD and TD groups. Solid and dashed blue lines show the average (with standard error of mean) decoding performance for each participant group. Based on cluster permutation testing, there were no significant differences between the participant groups.

Environmental sounds did not show a reliance on time-locked encoding, and no group differences were found based on cluster permutation testing (Fig. 6).

### Decoding performance for novel words

New words (pseudowords) also yielded good decoding performance with the time-locked models (Table 2). In the initial analysis with a wide lag window (20–420 ms), the amplitude envelope of pseudowords was decoded on average at 75% accuracy for the DLD group and at 80% accuracy for the TD group. Spectrogram decoding also performed well for the novel words (74% for both groups). The average decoding accuracy for the phoneme sequence was at 68% for the DLD group and at 69% for the TD group but only reached significance for a few participants (for this small set of pseudowords, the significance limit according to permutation tests was at ∼75%). In this initial analysis, there were no significant differences between groups.

**Figure 7.**
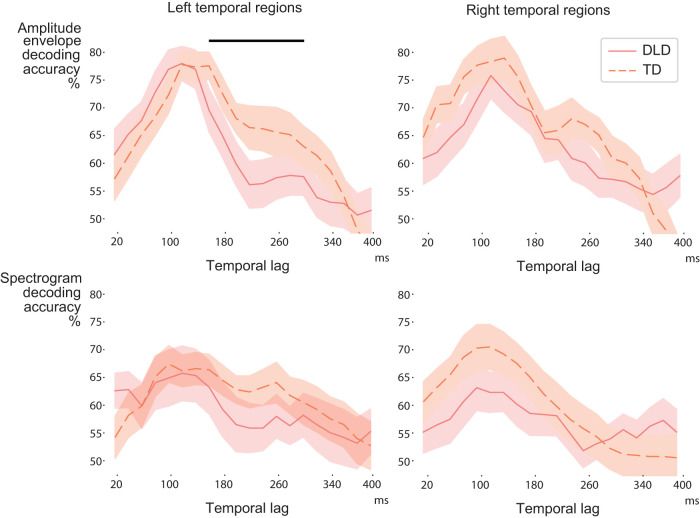
Envelope and spectrogram decoding for pseudowords in the left and right hemispheres in the DLD and TD groups. Solid and dashed red lines show the average (with standard error of mean) decoding performance for each participant group. The black bar denotes the largest observed cluster showing differences (TD > DLD) based on cluster permutation testing.

However, in the more detailed analysis of decoding at different temporal lags at 20 ms intervals, the TD group showed better decoding of pseudoword envelopes (largest cluster at ∼160–340 ms lag in bilateral temporal regions; *p* = 0.041; Fig. 7). When analyzing the left and right temporal regions separately, this difference reached significance only in the left hemisphere (largest cluster at ∼160–300 ms lag; *p* = 0.046). Spectrogram decoding did not reveal group differences. It is to be noted that these analyses on pseudowords are based on a small number of test stimuli (eight pseudowords) and thus are less reliable.

### Correlating decoding performance with behavioral performance

To investigate whether there is a relationship between behavioral performance and results of different decoding models, we calculated Pearson’s correlation coefficients with the two participant groups combined. There were some tentative correlations, but none of the correlations reached significance after correcting for multiple comparisons (*p* values given below are uncorrected). The overall decoding level did not correlate with one-back task performance for either spectrogram or envelope decoding of words or sounds or phoneme or semantic decoding of spoken words. However, semantic decoding of environmental sounds showed tentative correlation with one-back task performance for speech (*r* = 0.35; *p* = 0.044) and one-back task performance for environmental sounds (*r* = 0.45; *p* = 0.008). Speech envelope decoding at bilateral temporal regions at 160–340 ms lag showed a tentative correlation with the verbal reasoning index (*r *= 0.38; *p* = 0.026) and working memory index (*r* = 0.39; *p* = 0.024) of the Wechsler Intelligence Scale for Children-IV (WISC-IV). One-back task performance for speech also tentatively correlated with speech envelope decoding at bilateral temporal regions at 160–340 ms lag (*r* = 0.38; *p* = 0.025), as well as with verbal reasoning index (*r* = 0.35; *p* = 0.044) and working memory index (*r* = 0.45; *p* = 0.008) of the WISC-IV.

### Controlling for possible attentional differences

To verify that poor attention to the stimuli did not affect the group differences observed in speech decoding, we compared the decoding performance for pairs of participants, one from the DLD and one from the TD group who had similar one-back task performance. The decoding performance was averaged over spoken word and sound stimuli, participants performing under 50% were rejected, and the participants were arranged as pairs according to their one-back performance. This yielded equivalent performance on the one-back task but a significant difference in the decoding of speech envelope (at 160–340 ms lag window, using data from bilateral temporal regions, one-sided Wilcoxon signed rank test, *z* = −1.99.0; *p* = 0.024).

## Discussion

We investigated whether the cause of poorer spoken language comprehension and learning in DLD lies in the atypical cortical tracking of speech. The acoustic and phoneme contents of the spoken words were successfully decoded in both TD children and children with DLD. For both TD and DLD groups, decoding of acoustic features of spoken words was best at a lag of ∼100 ms between each time point of the unfolding spoken word and the corresponding time point in the cortical response, similarly to what has been observed earlier in adults (Nora et al., 2020). Cortical evoked activation thus followed speech closely in time. No such high reliance on time-locked encoding was observed for the environmental sounds. However, group differences emerged in the temporal details of cortical tracking of speech.

**Figure 8.**
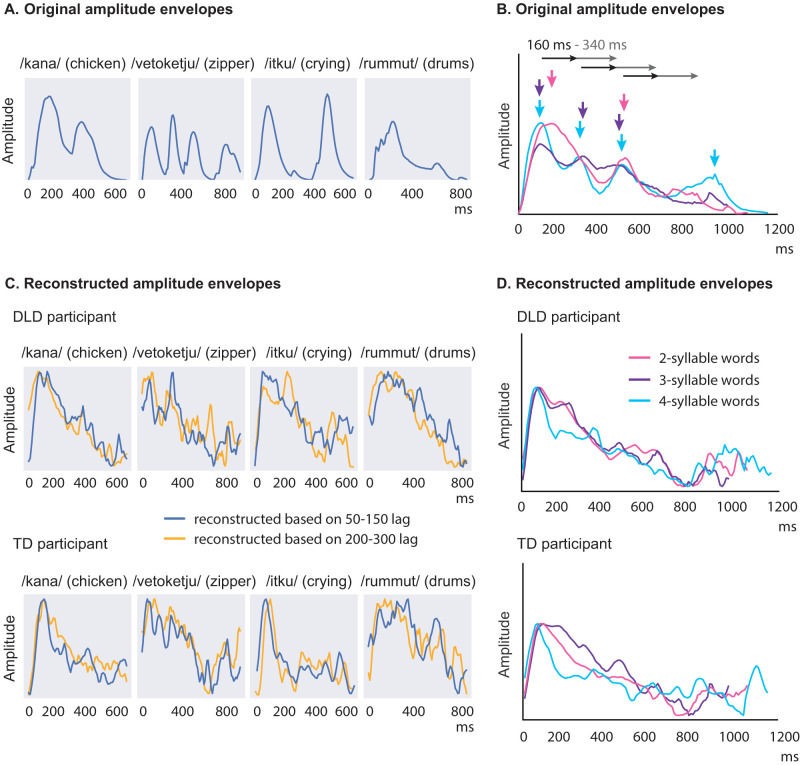
Illustration of the original and reconstructed amplitude envelopes of the spoken words. ***A***, Amplitude envelopes for example words. The overall syllable rhythm is clearly illustrated, but the amplitude envelope also carries information about phoneme identity, such as the voiceless stops /k/ and /t/ and the vibrations of the alveolar trill /r/, which are clearly visible in the envelope. ***B***, Average amplitude envelopes for words of different lengths (2, 3, and 4 syllables) in one stimulus set. The downward facing arrows mark the average syllable timings for stimuli of each syllable length. The black/gray arrow denotes the 160–340 ms lag, which showed better decoding of the spoken word amplitude envelope in the TD than DLD group; this corresponds roughly to between-syllable latency within the stimulus words. ***C***, Reconstructed amplitude envelopes for example words, based on data from bilateral temporal cortices at 50–150 and 200–300 ms lag, in one participant from each group (leave-one-out reconstruction). The reconstructions based on MEG data at both latencies most prominently highlight the first syllables (where the word stress lies in Finnish words) but also somewhat reflect the syllable rhythm in both participant groups. ***D***, Reconstructed amplitude envelopes averaged over two-, three-, and four-syllable words, based on data from bilateral temporal cortices at 20–420 ms lag, in one participant from each group (leave-one-out reconstruction).

The TD children displayed another, later peak in amplitude envelope decoding at ∼160–340 ms lag. This peak was significantly lower in the DLD group. The delayed decoding peak approximately corresponds to the lag between syllables for our stimulus words (average, 310 ms; range, 90–650 ms; Fig. 8). We propose that this difference in delayed decoding could reflect poorer maintenance of information in children with DLD, due to either faster decay of the auditory memory trace or stronger interference due to subsequent acoustic input. No differences between TD and DLD groups were observed in decoding environmental sounds, indicating some specificity of the observed differences for speech stimuli in the time-locked tracking of the amplitude envelope. However, some of the acoustic models performed at a significant level only for a few participants in both groups for environmental sounds, not allowing for comparisons between groups (Table 2). Thus, further study would be needed to draw conclusions on the specificity of the effects for speech stimuli.

The group differences in speech decoding do not seem to stem from attentional differences: the one-back task performance was not statistically different between the two groups. The overall success of decoding and the SNR of the cortical evoked responses were comparable between the two groups. Based on the success of decoding semantic features of the environmental sounds, the participants concentrated similarly on deriving sound meanings. Decoding the semantic features of spoken words was near the chance level, but this was true for both participant groups and is also in line with previous studies on adults (Simanova et al., 2010; Correia et al., 2015; Nora et al., 2020).

Nonetheless, we cannot completely rule out the possibility that the one-back memory task was somewhat more taxing for the participants with DLD, who typically have problems in verbal short-term memory, and that executive or attentional processes would contribute to the observed neural differences. In equivalence testing, the participant groups did not show equal performance in the one-back task: the DLD group seemed to show somewhat poorer performance, especially for speech. Near-significant correlations revealed a tentative connection between speech envelope decoding at 160–340 ms and one-back performance; however, they also indicated a relationship of the decoding differences to linguistic and working memory skills. Linguistic and working memory skills were, tentatively, also reflected in the one-back task. When equalizing for one-back performance, the differences in the delayed speech envelope decoding remained significant. In any case, the requirements of the one-back task resemble everyday speech processing: understanding spoken utterances requires identifying the individual words, activating their meanings in long-term memory, and retaining them in short-term memory to integrate them to the following words in the sentence. In this way, the observed impairments in the maintenance of speech representations in DLD could be expected to show in everyday speech processing of continuous speech.

### The low-level deficit hypothesis revisited

The early decoding peak at ∼100 ms, similar in the TD and DLD groups, was observed both for the amplitude envelope and spectrogram which contain overlapping information (Obleser et al., 2012; Teng et al., 2019). This is approximately the time frame in which categorical neural organization for phonemes transiently emerges (Ostroff et al., 1998; Tremblay et al., 2003; Chang et al., 2010), and tracking of speech acoustics at this latency is coupled with phonological representations (Nora et al., 2020). Linguistic representations are thought to emerge directly from tuning to the complex spectrotemporal acoustic feature characteristic of different phonemes (Mesgarani et al., 2014; de Heer et al., 2017). The current results suggest that this initial analysis of translating the acoustic stream into phoneme representations in the temporal cortical regions is not impaired or slower in children with DLD.

Our current results are compatible with earlier behavioral studies of DLD reporting, for example, impaired performance in discrimination of amplitude modulations (Cumming et al., 2015; Richards and Goswami, 2015; Caccia and Lorusso, 2019), but they call for a new interpretation of the previous findings. In those studies, children with DLD performed more poorly, for example, in deciding which “ba” syllable or which tone had a longer rise time or longer duration and determining where the stress lies in multisyllabic words or syllable sequences (Cumming et al., 2015; Richards and Goswami, 2015). Based on the present results of similar decoding in TD and DLD groups at ∼100 ms lag but a salient group difference at longer lags, we propose that those earlier observations of impaired performance may not be tied to impaired encoding of the amplitude modulations per se but, instead, might reflect impaired maintenance of cortical activity representing the amplitude modulations. This could impair the necessarily delayed comparisons of amplitude rise times or intensities of two syllables over time and, more crucially, speech processing.

### Impairments in maintaining acoustic–phonetic representations in DLD show bilaterally in the temporal cortices

Time-locked tracking of the acoustic content in bilateral temporal areas, with no clear lateralization, is consistent with previous studies and compatible with the view that acoustic–phonetic processing is bilaterally implemented (de Heer et al., 2017; Brodbeck et al., 2018; Nora et al., 2020). Previous MEG studies on spoken word/pseudoword repetition have highlighted left-hemispheric differences between language-impaired and TD children and adults in short-term memory of word-level information (Helenius et al., 2009, 2014; Nora et al., 2021). The present paradigm that investigated the tracking of subword-level acoustic information, however, revealed bilateral differences between DLD and TD children in the tracking of familiar and new words in evoked responses of temporal areas (Figs. 5, 7). When the decoding was tested separately in each hemisphere, group differences at longer latencies reached significance in the right temporal regions for real (familiar) words and in the left temporal regions for pseudowords (new words).

The left and right auditory regions have been suggested to have somewhat different roles in the processing of speech envelope information. To simplify, it has been suggested that the coarser (syllable and word rate) modulations are processed with a right-hemispheric preference and finer (phoneme rate) modulations with a bilateral or left-hemispheric preference (Poeppel, 2003; Boemio et al., 2005; Abrams et al., 2008; Vanvooren et al., 2014; Ylinen et al., 2015). The amplitude envelope of speech is dominated by low-frequency modulations, which are more strongly tracked in the right hemisphere, and impairments in speech envelope tracking in dyslexic children have also been observed more prominently in the right temporal regions (Abrams et al., 2009; Molinaro et al., 2016; Di Liberto et al., 2018). The current results could be compatible with more rightward impairment for speech tracking also in DLD. However, with this first attempt at uncovering deficient speech tracking in DLD, conclusions on hemispheric differences are not yet warranted without further studies.

### Possible consequences of impaired speech tracking for processing and learning words

When we hear a word, we hear a sequence of sounds spread across time. Multiple lexical candidates are activated in long-term memory while an auditory word is being perceived, until a single item is selected and the word recognized (Marslen-Wilson and Tyler, 1980; Luce, 1986; McClelland and Elman, 1986; Marslen-Wilson, 1987; Gaskell and Marslen-Wilson, 1997). Thus, a detailed echoic memory representation of speech sounds needs to be stored temporarily until enough information is accumulated for identifying the word (Calabrese, 2012; Gwilliams et al., 2018). Simultaneously, the perception of earlier sounds is reassessed with later acoustic input. Children with DLD show deficits even in recognizing familiar spoken words (Dollaghan, 1998; Mainela-Arnold et al., 2008). The impaired maintenance of sublexical acoustic–phonetic information observed here for children with DLD likely results in problems in activating the correct word-level representations for familiar words in long-term memory based on incoming speech input. It may potentially lead to increased vulnerability to competing words, as has been observed in behavioral studies (Mainela-Arnold et al., 2008; McMurraya et al., 2019), when the competition between lexical candidates is more difficult to solve due to faster decay of auditory traces.

One of the most robust findings in DLD is impaired phonological memory, reflected, for example, in pseudoword repetition (Bishop et al., 1996; Graf Estes et al., 2007). Impaired phonological memory is thought to be a bottleneck for learning new words (Baddeley, et al., 1998; Adams and Gathercole, 2000), impairing language learning in children and adults with DLD (Attout et al., 2020; Jackson et al., 2021). If the children with DLD are unable to accurately represent earlier parts of the word in memory, as suggested by the current results, tentatively, they might not be able to properly integrate across word segments to form new lexical representations. This would particularly impair the cortical representations for novel word forms. This possible interpretation of the current findings should be investigated in learning paradigms, where the impaired tracking could be directly related to the integration of syllable-level information for forming new lexical representations.

It is possible that a similar impairment in maintaining the speech representations that was observed here for individual words would also be observed in the processing of continuous speech. Listeners with DLD have been shown to have difficulties in maintaining cortical representations from one spoken word to the next (Helenius et al., 2009, 2014). Cortical tracking of amplitude modulations should also be studied for continuous speech to uncover whether its deficits in DLD also contribute to problems in sentence-level processing. Similarly as was done in this study for individual words, a response function for speech amplitude envelope could be constructed for continuous speech by kernel estimation (as in Brodbeck et al., 2018) to investigate this possibility. Investigating speech tracking across different frequency ranges could also further shed light on the potential hemispheric differences in the observed impairment in DLD.
